# Trends in sociodemographic factors associated with human papillomavirus vaccine hesitancy in the United States: 2010–2022

**DOI:** 10.1002/cncr.70636

**Published:** 2026-09-28

**Authors:** Dina K. Abouelella, Mrudula Nair, Mary A. Gerend, Olivia Aspiras, Nosayaba Osazuwa‐Peters, Eric Adjei Boakye

**Affiliations:** ^1^ Department of Head and Neck Surgery and Communication Sciences Duke University School of Medicine Durham North Carolina USA; ^2^ Department of Public Health Sciences Henry Ford Health System Detroit Michigan USA; ^3^ Department of Behavioral Sciences and Social Medicine Florida State University College of Medicine Tallahassee Florida USA; ^4^ Charles Stewart Mott Department of Public Health College of Human Medicine Michigan State University Flint Michigan USA; ^5^ Duke Cancer Institute Duke University Durham North Carolina USA; ^6^ Henry Ford Health + Michigan State University Health Sciences Detroit Michigan USA; ^7^ Department of Otolaryngology–Head and Neck Surgery Henry Ford Health System Detroit Michigan USA; ^8^ Department of Epidemiology and Biostatistics Michigan State University College of Human Medicine East Lansing Michigan USA

**Keywords:** HPV vaccine, human papillomavirus, National Immunization Survey–Teen, sociodemographic characteristics, vaccination, vaccine hesitancy

## Abstract

**Background:**

Previous studies suggest that rates of human papillomavirus (HPV) vaccine hesitancy vary by adolescent and parent sociodemographic characteristics. This study assessed trends in the characteristics of parents who did not intend to have their child vaccinated for HPV in the United States.

**Methods:**

The authors used data from the 2010–2022 National Immunization Survey–Teen among adolescents who had received zero doses of the HPV vaccine. Vaccine hesitancy was assessed with the question “How likely is it that your teen will receive HPV shots in the next 12 months?” Parents who responded with “not too likely, “not likely at all,” or “not sure/don't know” were deemed vaccine hesitant. Joinpoint regression estimated yearly increases and/or decreases in vaccine hesitancy by adolescent and mother characteristics through annual percent changes.

**Results:**

A total of 204,196 unvaccinated adolescents were included in the study, 66.0% had parents who were classified as HPV vaccine hesitant. Overall, the proportion of adolescents with mothers who were HPV vaccine hesitant declined from 72% in 2010 to 61% in 2022. Among parents with female children, HPV vaccine hesitancy decreased significantly from 2010 to 2012, increased significantly from 2012 to 2018 followed by a significant decrease from 2018 to 2022. Among parents with male children, HPV vaccine hesitancy decreased significantly from 2010 to 2012 and remained stable from 2012 to 2022. Parents with children of all racial and ethnic groups experienced significant decreases in HPV vaccine hesitancy from 2010 to 2012 and then remained stable from 2012 to 2022.

**Conclusions:**

This study emphasizes the need to dispel misconceptions and create awareness about HPV vaccine across all strata of the society.

## Abstract 2

Trends in the characteristics of parents who did not intend to have their child vaccinated for human papillomavirus in the United States were examined. There was a decrease in vaccine hesitancy from 2010 to 2012 for all socioeconomic characteristics examined in the study but a stable trend for most socioeconomic characteristics from 2012 to 2020.

## INTRODUCTION

In 2019, the World Health Organization (WHO) labeled vaccine hesitancy as one of the top 10 health threats.^1^ The WHO defines vaccine hesitancy as a delay in acceptance or refusal of safe vaccines despite availability of vaccination services.^2^ Progress in human papillomavirus (HPV) vaccine uptake in the United States and many other developed countries has been significantly impacted by vaccine hesitancy.^3^, ^4^ Hesitancy differs across time, place, and vaccine type, and is affected by several multilevel factors, chief among them which are fear, misconceptions, and mistrust.^2^ In the United States, at least one in three families are HPV vaccine hesitant,^5^ making hesitancy one of the strongest predictors of poor HPV uptake.

Approximately 80 million people (25% of the United States population) are currently infected with at least one strain of HPV,^6^ and the virus is associated with nearly 34,800 new cases of cancer each year.^7^ HPV vaccination is the best primary prevention strategy for most HPV‐associated cancers, and current evidence suggests that 92% of HPV‐attributable cancers could be prevented with vaccination.^8^ Routine HPV vaccination at age 11 or 12 years (starting as young as age 9, with catch up through age 26 years) was recommended initially for females in 2006. Although males received a provisional recommendation for HPV vaccination in 2009, routine recommendation was not approved until 2011.^9^, ^10^ Unfortunately, however, vaccination rates remain below the Healthy People 2030 goal of 80% completion for adolescents.^11^ In 2023, only 61.4% of eligible adolescents were up to date on the HPV vaccine series which was similar to the previous year’s rate of 62.4%.^12^ HPV vaccine hesitancy is a nationwide concern and is strongly associated with under‐vaccination as well as vaccine refusal.^13^

Rates of HPV vaccine hesitancy differ based on the social and demographic characteristics of adolescents and their parents, including the child’s gender, race/ethnicity, poverty level, and whether they received a provider recommendation, as well as the mother’s marital status, educational level, and health insurance status.^13^, ^14^, ^15^ However, it is unknown if the sociodemographic factors or characteristics associated with vaccine hesitancy have changed over time. We investigated trends in the sociodemographic characteristics of HPV vaccine–hesitant parents and their child(ren) in the United States from 2010 to 2022.

## MATERIALS AND METHODS

### Data source and design

We used data from the National Immunization Survey−Teen (NIS–Teen) spanning from 2010 to 2022. The NIS–Teen was performed by the Center for Disease Control’s National Center for Immunization and Respiratory Diseases. The NIS–Teen is an annual, cross‐sectional survey, using two stages to collect immunization data for adolescents 13–17 years old. The NIS–Teen uses random‐digit dialing to survey households with age‐eligible adolescents and, after verbal consent is obtained, to contact vaccination providers to obtain vaccination information. If an adolescent has multiple vaccine providers, a single immunization history is constructed. Details of NIS–Teen survey sampling, data collection, and weighting operations have been described previously.^16^ The NIS–Teen was approved by the National Center for Health Statistics research ethics review board. NIS–Teen data are deidentified and publicly available; therefore, the secondary data analysis conducted in this study was exempt from institutional review board approval.

### Measures

The outcome of interest was HPV vaccine hesitancy, which was assessed with the question, “How likely is it [your teen] will receive HPV shots in next 12 months?” This question was asked of parents/guardians of adolescents who had not received any HPV vaccine doses. Parents who selected “not too likely,” “not likely at all,” or “not sure/don’t know” were classified as being HPV vaccine hesitant. Parents who selected “very likely” or “somewhat likely” were classified as not hesitant. Hesitancy classification was based on what has been used in prior literature.^13^, ^15^, ^17^

Adolescent characteristics included age in years (13, 14, 15, 16, or 17), sex (female or male), race/ethnicity (non‐Hispanic White, non‐Hispanic Black, Hispanic, or non‐Hispanic other), number of doctor’s visits in the past 12 months (none, one, two to three, and ≥four), poverty level (recorded as annual income “above poverty >$75,000” or “below poverty < $75,000,” which is based on the United States’ Census family poverty levels), whether the adolescent received a recommendation to get the HPV vaccine from a health care provider (yes or no), and geographic region (Northeast, Midwest, South, or West). Parent/guardian characteristics included mother’s age in years (≤34, 35–44, or ≥45), mother’s marital status (married or not married), mother’s educational level (less than high school, high school graduate, some college, or college graduate or higher), and the number of children under 18 years of age living in the household (one, two to three, or four or more).

### Statistical analysis

Descriptive statistics were used to analyze respondents’ characteristics. Differences between adolescent and parent characteristics and HPV vaccine hesitancy status were examined through χ^2^ testing. Next, multivariable logistic regression analysis was performed to examine the association between adolescent, maternal characteristics and year of survey, and vaccine hesitancy. These analyses were performed using SAS 9.4 (SAS Institute Inc, Cary, North Carolina) survey‐specific procedure, which incorporates survey sampling weights and stratum to account for the complex sampling design used in the NIS–Teen and to provide representative estimates of the US population. Statistical tests were two tailed and *p* < .05 was considered statistically significant.

Trends in adolescent and parent characteristics associated with vaccine hesitancy were examined using joinpoint regression, a variant of log‐linear regression.^18^ Joinpoint program (version 4.9.0.0, Statistical Methodology and Applications Branch, Surveillance Research Program, National Cancer Institute) was used to estimate joinpoint regression models to determine significant changes in vaccine hesitancy during the 13‐year span. Joinpoint regression models determined the starting and ending years of increases/decreases (joinpoints) and then estimated the annual percentage change (APC) and 95% confidence intervals (CI) based on a regression model between the two joinpoint years. The final joinpoint models were based on log‐transformed percentages to better ensure normality of residuals. The model with the least number of joinpoints (two in this case) necessary to effectively characterize trends was based on the permutation test method. The permutation test method determined the model with the fewest number of joinpoints necessary to effectively characterize trends for each subgroup.

## RESULTS

The total number of adolescents who were eligible to receive the HPV vaccine was 518,806; of whom 300,471 (57.9%) had received at least one dose of the vaccine. A total of 218,335 unvaccinated adolescents were included in the study from 2010 to 2022. Among unvaccinated adolescents, 134,717 (66.0%) had parents who were classified as HPV vaccine hesitant. Overall, 59.4% of the unvaccinated adolescents were males, 66.3% were non‐Hispanic White adolescents, 86.8% lived above the poverty level, 39.2% resided in the South, and 64.0% had not received a provider recommendation for the HPV vaccine (Table S1). Most mothers were ≥35 years old (92.5%) and married (73.9%) (Table S1). Table 1 summarizes the characteristics of the respondents stratified by HPV vaccine hesitancy status. In bivariate analyses, statistically significant differences were observed between the hesitant and nonhesitant cohorts for all sociodemographic variables (*p* < .05). A higher proportion of parents with children who were age 17 years were HPV vaccine hesitant than those with children who were age 13 years (69.2% vs. 60.8%). Similarly, a higher proportion of parents were HPV vaccine hesitant if they had children who were non‐Hispanic White (69.8%) versus Hispanic (54.8%), and live above poverty level (67.2%) versus below the poverty level (54.4%). A higher proportion of mothers were HPV vaccine hesitant if they were ≥45 years of age (66.4%) versus ≤34 years (57.8%), married (68.1%) versus not married (59.2%), and had college degree or higher (67.4%) versus high school diploma or less (52.1%). As of 2022, HPV vaccine hesitancy was highest among parents with children who were age 17 years (67.7%), non‐Hispanic White (67.3%), lived above the poverty level (62.0%), and mothers who were married (64.1%), and had some college education or had college degree (63.0%) (data used in sentence not in Table 1).

**TABLE 1:** Characteristics of study respondents by HPV vaccine hesitant status: National Immunization Survey–Teen, 2010–2022 (unweighted *n* = 218,335).

	HPV vaccine hesitant, No. (%)	HPV vaccine nonhesitant, No. (%)	*p*
Adolescent			
Age at time of survey, years			<.0001
13	27,668 (60.8)	17,425 (39.2)	
14	27,690 (64.3)	15,221 (35.7)	
15	26,648 (64.9)	13,563 (35.1)	
16	27,196 (67.6)	12,363 (32.4)	
17	25,515 (69.2)	10,907 (30.8)	
Sex			<.0001
Female	53,067 (63.1)	29,831 (36.9)	
Male	81,650 (66.6)	39,648 (33.4)	
Race/ethnicity			<.0001
Non‐Hispanic White	93,627 (69.8)	41,768 (30.2)	
Non‐Hispanic Black	11,818 (60.6)	7623 (39.4)	
Hispanic	16,492 (54.8)	13,054 (45.2)	
Non‐Hispanic other	12,780 (64.1)	7034 (35.9)	
Poverty status			<.0001
Above poverty	11,1867 (67.2)	54,488 (32.8)	
Below poverty	13,879 (54.4)	11,333 (45.6)	
Number of doctor’s visits in the past 12 months			<.0001
≥4	22,957 (63.5)	12,727 (36.5)	
2–3	44,010 (63.6)	24,645 (36.4)	
1	41,707 (67.2)	20,122 (32.8)	
None	25,114 (66.4)	11,507 (33.6)	
Census region			<.0001
Northeast	21,689 (64.5)	12,712 (35.5)	
Midwest	30,164 (67.6)	14,037 (32.4)	
South	51,955 (64.4)	28,088 (35.6)	
West	30,909 (64.6)	14,642 (35.4)	
Provider recommendation			<.0001
Yes	41,923 (59.3)	28,912 (40.7)	
No	87,860 (68.4)	37,802 (31.6)	
Mother			
Age, years			<.0001
≤34	8951 (57.8)	6241 (42.2)	
35–44	58,535 (65.2)	29,760 (34.8)	
≥45	67,231 (66.4)	33,478 (33.6)	
Marital status			<.0001
Married	102,737 (68.1)	48,086 (31.9)	
Not married	31,980 (59.2)	21,393 (40.8)	
Education			<.0001
College graduate or higher	62,001 (67.4)	30,592 (32.6)	
Some college	40,363 (68.1)	18,505 (31.9)	
High school graduate	23,288 (64.3)	12,598 (35.7)	
Less than high school	9065 (52.1)	7784 (47.9)	
No. of children under age 18			.0015
1	53,663 (64.9)	27,723 (35.1)	
2 or 3	66,608 (64.9)	35,576 (35.1)	
4 or more	14,446 (67.1)	6180 (32.9)	

*Note:* Percents for hesitant and nonhesitancy are reported as row percents.

Abbreviation: HPV, human papilloma virus.

Overall, the proportion of adolescents with mothers who were HPV vaccine hesitant declined from 72% in 2010 to 61% in 2022. Joinpoint analyses examined trends in HPV vaccine hesitancy stratified by adolescent age, gender, race/ethnicity and poverty status, and mother’s marital status and education. HPV vaccine hesitancy decreased significantly by an average of 10% per year from 2010 to 2012 among parents with 13‐year‐old children (APC, –9.8%; 95% CI, –10.8% to –9.0%; *p* < .001), remained stable from 2012 to 2015, and then decreased significantly from 2015 to 2022 by 2% annually (APC, –2.1%; 95% CI, –2.6% to –1.8%; *p* < .001) (Figure 1A; Table 2). The same pattern (HPV vaccine hesitancy decreased significantly from 2010 to 2012, remained stable from 2012 to 2015, and decreased significantly from 2015 to 2022) was observed for parents with children 14–15 years old (Figure 1A; Table 2). Among parents with female children, HPV vaccine hesitancy decreased significantly by 5% annually (APC = –4.5%; 95% CI, –7.9% to –0.3%; *p* = .030) from 2010 to 2012, increased significantly by 1% annually from 2012 to 2018 (APC, 1.2%; 95% CI, 0.3%–4.8%; *p* = .015), followed by a significant decrease of 3% annually from 2018 to 2022 (APC, –2.5%; 95% CI, –7.5% to –0.9%; *p* = .006) (Figure 1B; Table 2). Among parents with male children, HPV vaccine hesitancy decreased significantly by 6% annually (APC, –6.2%; 95% CI, –8.0% to –3.9%; *p* < .001) from 2010 to 2012 and remained stable from 2012 to 2022 (Figure 1B; Table 2). Parents with children of all racial and ethnic groups experienced significant decreases in HPV vaccine hesitancy from 2010 to 2012 (range from –6.8% to –5.3% per year; *p* < .001) (Figure 1C; Table 2). Among parents with non‐Hispanic White, non‐Hispanic Black, and Hispanic adolescents, there were no significant changes from 2012 to 2022 (Figure 1C; Table 2). HPV vaccine hesitancy for parents of adolescents who lived above the poverty level had a significant annual decrease in vaccine hesitancy from 2010 to 2012 by 6% (APC, –5.6%; 95% CI, –6.5% to –4.5%; *p* < .001), remained stable from 2012 to 2018, and then decreased significantly from 2018 to 2022 by 2% annually (APC = –1.8%; 95% CI, –3.2% to –1.1%; *p* < .001) (Figure 1D; Table 2). HPV vaccine hesitancy for parents of adolescents who lived below the poverty level had a significant annual decrease in vaccine hesitancy from 2010 to 2012 (APC, –5.8%; 95% CI, –9.2% to –1.7%; *p* = .001) and then remained stable from 2012 to 2022 (Figure 1D; Table 2).

**FIGURE 1:**
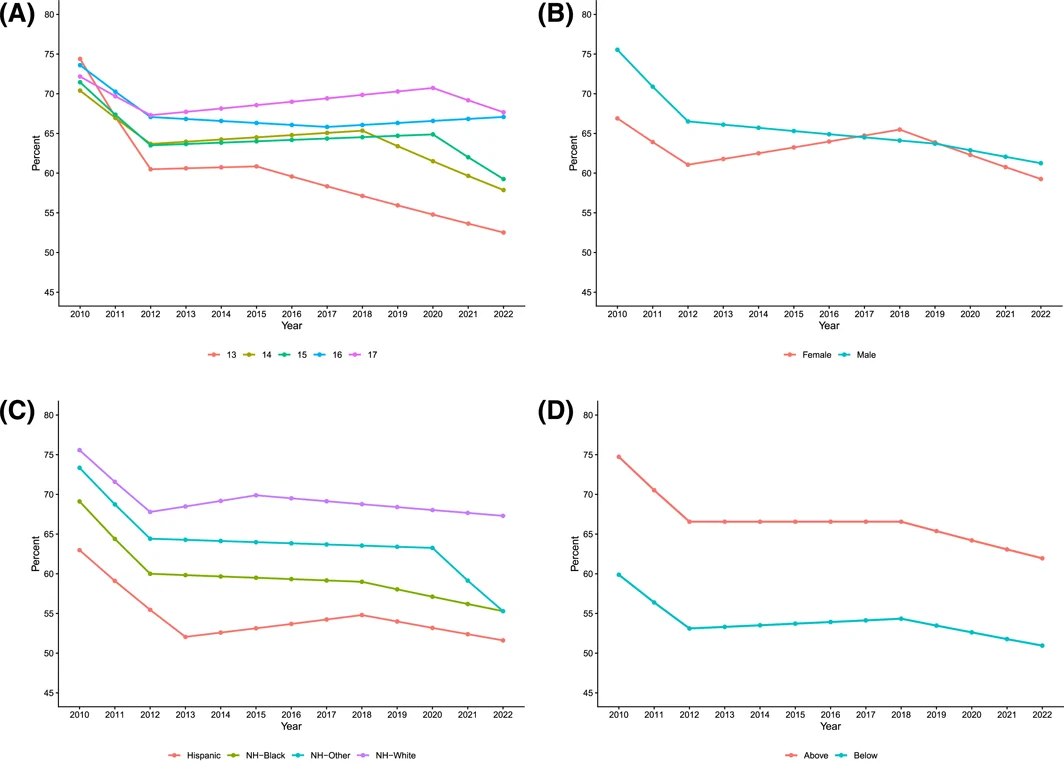
Joinpoint analysis of the proportion of parents who are HPV vaccine hesitant in the United States, 2010–2022. (A) Adolescent age at time of survey in years. (B) Adolescent gender. (C) Adolescent race/ethnicity. (D) Adolescent poverty status. Data is from the National Immunization Survey–Teen. NHBlack indicates non‐Hispanic Black; NHOther, non‐Hispanic other; NHWhite, non‐Hispanic White.

**TABLE 2:** APC in HPV vaccine hesitancy.

	APC	95% CI		*p*
		Lower bound	Upper bound	
Adolescent age at time of survey, years				
13				
2010–2012	–9.8	–10.8	–9.0	<.001
2012–2015	0.2	–0.7	0.9	.782
2015–2022	–2.1	–2.6	–1.8	<.001
14				
2010–2012	–4.9	–7.7	–1.6	<.001
2012–2018	0.4	–0.4	3.2	.198
2018–2022	–3.0	–7.3	–1.4	.003
15				
2010–2012	–5.7	–8.3	–1.9	<.001
2012–2020	0.2	–0.2	3.5	.156
2020–2022	–4.4	–9.3	–1.0	.004
16				
2010–2012	–4.5	–6.0	–2.7	<.001
2012–2017	–0.4	–1.3	1.0	.452
2017–2022	0.4	–1.6	2.5	.393
17				
2010–2012	–3.4	–5.3	–0.9	<.001
2012–2020	0.6	0.2	2.3	.021
2020–2022	–2.2	–4.9	0.2	.093
Adolescent gender				
Female				
2010–2012	–4.5	–7.9	–0.3	.030
2012–2018	1.2	0.3	4.8	.015
2018–2022	–2.5	–7.5	–0.9	.006
Male				
2010–2012	–6.2	–8.0	–3.9	<.001
2012–2019	–0.6	–1.5	1.5	.460
2019–2022	–1.3	–4.8	0.5	.133
Adolescent race/ethnicity				
Non‐Hispanic White				
2010–2012	–5.3	–7.3	–2.9	<.001
2012–2015	1.0	–0.4	2.7	.112
2015–2022	–0.5	–4.2	0.2	.074
Non‐Hispanic Black				
2010–2012	–6.8	–10.8	–2.5	<.001
2012–2018	–0.3	–1.8	3.8	.814
2018–2022	–1.6	–8.3	1.2	.167
Hispanic				
2010–2013	–6.2	–10.2	–3.6	<.001
2013–2018	1.0	–0.4	4.5	.079
2018–2022	–1.5	–6.2	0.2	.061
Non‐Hispanic other				
2010–2012	–6.3	–8.5	–2.8	<.001
2012–2020	–0.2	–0.7	1.8	.990
2020–2022	6.5	–10.0	–2.2	<.001
Adolescent poverty status				
Above poverty				
2010–2012	–5.6	–6.5	–4.5	<.001
2012–2018	0.0	–0.3	0.7	.885
2018–2022	–1.8	–3.2	–1.1	<.001
Below poverty				
2010–2012	–5.8	–9.2	–1.7	.001
2012–2018	0.4	–0.9	3.8	.243
2018–2022	–1.6	–7.2	0.9	.113
Mothers’ marital status				
Married				
2010–2012	–5.2	–6.6	–3.3	<.001
2012–2018	0.2	–0.2	1.7	.287
2018–2022	–1.4	–3.9	–0.5	.005
Not married				
2010–2012	–5.9	–7.5	–3.7	<.001
2012–2018	–0.3	–0.9	1.1	.788
2019–2022	–1.3	–3.4	–0.1	.040
Mothers’ education level				
College graduate or higher				
2010–2012	–6.1	–8.5	–2.8	<.001
2012–2020	–0.2	–0.9	2.7	.893
2020–2022	–3.6	–8.0	–0.3	.024
Some college				
2010–2012	–5.5	–7.4	–3.5	<.001
2012–2015	–0.6	–0.8	2.0	.480
2015–2022	–1.1	–3.8	–0.1	.042
High school graduate				
2010–2013	–4.8	–7.5	–3.4	<.001
2013–2017	1.5	0.4	3.6	.004
2017–2022	–1.5	–3.5	–0.8	<.001
Less than high school				
2010–2012	–7.2	–10.9	–2.8	.002
2012–2020	–0.1	–2.0	3.2	.865
2020–2022	3.4	–1.9	9.1	.413

Abbreviations: APC, annual percent change; HPV, human papilloma virus.

Among mothers of adolescents who were married, HPV vaccine hesitancy decreased significantly by 5% per year (APC, –5.2%; 95% CI, –6.6% to –3.3%; *p* < .001) from 2010 to 2012, remained stable from 2012 to 2018, and then decreased significantly from 2018 to 2022 by 1% per year (APC, –1.4%; 95% CI, –3.9% to –0.5%; *p* = .005) (Figure 2A; Table 2). Among mothers of adolescents who were not married, HPV vaccine hesitancy decreased significantly by 6% per year (APC, –5.9%; 95% CI, –7.5% to –3.7%; *p* < .001) and then remained stable from 2012 to 2022 (Figure 2A; Table 2). The average HPV vaccine hesitancy decreased by 6% per year 2010 to 2012 for mothers who had a college degree or higher (APC, –6.1%; 95% CI, –8.5% to –2.8%, *p* < .001) and again by 4% from 2020 to 2022 (APC, –3.6%; 95% CI, –8.0% to –0.3%, *p* = .024) (Figure 2B; Table 2). The average HPV vaccine hesitancy increased by 6% per year 2010 to 2012 for mothers who had some college/associate degree (APC, –5.5%; 95% CI, –7.4% to –3.5%, *p* < .001) but decreased by 1% from 2015 to 2022 (APC, –1.1%; 95% CI, –3.8% to –0.1%, *p* = .042) (Figure 2B; Table 2). The average HPV vaccine hesitancy decreased by 5% per year 2010 to 2013 for mothers who had high school diploma (APC, –4.8%; 95% CI, –7.5% to –3.4%, *p* < .001), decreased by 2% per year from 2013 to 2017 (APC, –1.5%; 95% CI, –0.4% to –3.6%, *p* = .004), and decreased by 2% from 2017 to 2022 (APC, –1.5%; 95% CI, –3.5% to –0.8%, *p* < .001) (Figure 2B; Table 2). The average HPV vaccine hesitancy increased by 7% per year 2010 to 2012 for mothers who had less than high school diploma (APC, –7.2%; 95% CI, –10.9% to –2.8%, *p* = .002), but remained stable from 2012 to 2022 (Figure 2B; Table 2).

**FIGURE 2:**
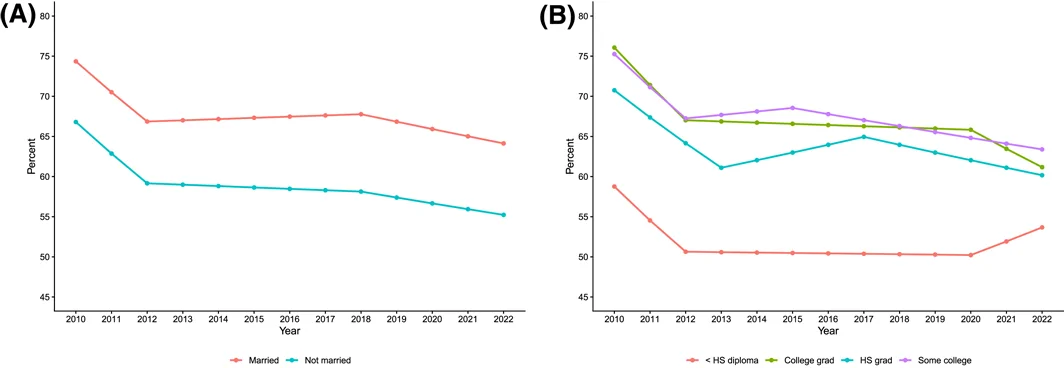
Joinpoint analysis of the proportion of parents who are HPV vaccine hesitant in the United States, 2010–2022. (A) Mother’s marital status. (B) Mother’s education level. Data is from the National Immunization Survey–Teen. CollegeGrad indicates college graduate or higher; HSDiploma, high school graduate; LessThanHS, less than high school graduate; SomeCollege, some college.

Results from the multivariable logistic regression estimating adolescent and mother's characteristics and HPV vaccine hesitancy status are presented in Table 3. In the adjusted regression analyses, parents of adolescents who were non‐Hispanic Black (adjusted odds ratio [aOR], 0.74; 95% CI, 0.70–0.78) or Hispanic (aOR, 0.63; 95% CI, 0.59–0.66) versus non‐Hispanic White; lived below the poverty level (aOR, 0.71; 95% CI, 0.67–0.75) versus above had lower odds of being vaccine hesitant. On the other hand, parents of adolescents who did not received a provider recommendation for the vaccine (aOR, 1.60; 95% CI, 1.54–1.66) had higher odds of being vaccine hesitant than those that did receive recommendation. Mothers with less than a high school diploma (aOR, 0.70; 95% CI, 0.65–0.75) versus college degree or higher, and not married (aOR, 0.81; 95% CI, 0.78–0.85) versus married had lower odds of being vaccine hesitant.

**TABLE 3:** Adolescent and mother’s factors associated with HPV vaccine hesitancy, National Immunization Survey–Teen, 2010–2022 (unweighted *n* = 204,196).

	aOR (95% CI)
Adolescent	
Age at time of survey, years	
13	Reference
14	1.17 (1.11–1.23)
15	1.22 (1.16–1.29)
16	1.36 (1.29–1.44)
17	1.44 (1.36–1.53)
Sex	
Female	Reference
Male	1.03 (0.99–1.06)
Race/ethnicity	
Non‐Hispanic White	Reference
Non‐Hispanic Black	0.74 (0.70–0.78)
Hispanic	0.63 (0.59–0.66)
Non‐Hispanic other	0.82 (0.77–0.87)
Poverty status	
Above poverty	Reference
Below poverty	0.71 (0.67–0.75)
No. of doctor’s visits in the past 12 months	
≥4	Reference
2–3	1.01 (0.96–1.06)
1	1.17 (1.11–1.24)
None	1.19 (1.12–1.26)
Census region	
Northeast	Reference
Midwest	1.09 (1.04–1.14)
South	1.03 (0.99–1.08)
West	1.09 (1.03–1.16)
Provider recommendation	
Yes	Reference
No	1.60 (1.54–1.66)
Mother	
Age, years	
≤34	Reference
35–44	1.13 (1.05–1.21)
≥45	1.07 (1.00–1.15)
Marital status	
Married	Reference
Not married	0.81 (0.78–0.85)
Education level	
College graduate or higher	Reference
Some college	1.10 (1.06–1.15)
High school graduate	0.95 (0.91–1.00)
Less than high school	0.70 (0.65–0.75)
No. of children under age 18	
1	Reference
2 or 3	1.04 (1.00–1.08)
≥4	1.28 (1.20–1.37)
Survey year	
2010	Reference
2011	0.81 (0.74–0.88)
2012	0.72 (0.67–0.78)
2013	0.71 (0.66–0.77)
2014	0.80 (0.74–0.87)
2015	0.79 (0.73–0.85)
2016	0.78 (0.72–0.84)
2017	0.81 (0.74–0.88)
2018	0.84 (0.77–0.92)
2019	0.76 (0.69–0.83)
2020	0.76 (0.70–0.83)
2021	0.67 (0.62–0.74)
2022	0.70 (0.64–0.76)

Abbreviations: aOR, adjusted odds ratio; HPV, human papilloma virus.

## DISCUSSION

The objective of this study was to assess trends in the sociodemographic characteristics of parents who do not intend to initiate the HPV vaccine series for their adolescent son or daughter from 2010 to 2022. According to our findings, HPV vaccine hesitancy remains high in the United States. Our study is different from any other in that it assesses the change in vaccine hesitancy by sociodemographic characteristics over a period of 13 years, compared to previous studies that assessed vaccine hesitancy for only a few years (1–3 years)^13^, ^14^, ^15^, ^19^ or studied reasons for vaccine hesitancy.^15^, ^17^, ^20^, ^21^ This provides valuable information for health care providers, public health officials, and law makers to better understand which groups should be targeted to reduce HPV vaccine hesitancy and increase HPV vaccine uptake.

We found that the proportion of parents of male adolescents who were vaccine hesitant declined significantly from 2010 to 2012 and then stabilized from 2012 to 2018. On the other hand, the proportion of vaccine hesitant parents with adolescent daughters did decrease over the 13‐year study period. Although parents of adolescent males initially showed higher decline in HPV vaccine hesitancy than those of females, the trend remained stable the last 10 years. HPV vaccination was initially targeted toward females, advertised for cervical cancer prevention, and was not approved for males until 2009, 3 years after its initial Food and Drug Administration approval in 2006. These factors have resulted in parents of males having lower HPV vaccine awareness and uptake compared to females.^22^, ^23^, ^24^, ^25^, ^26^ However, the initial significant decline in vaccine hesitancy among parents of adolescent males between 2010 and 2012 may be attributed to the inclusion of males in the national HPV vaccination program efforts which began in 2011 when routine HPV vaccination for males was approved by the Advisory Committee on Immunization Practices.^27^ This temporal alignment suggests that national policy changes may have positively influenced parental attitudes during this period coincide with our findings but has diminished for parents with male adolescents. Further investigation is required to understand the reasons for this change. Further research is also needed to examine potential strategies to address the underlying factors in parental vaccine hesitancy, especially among parents of male adolescents. For example, researchers could develop and evaluate gender‐targeted interventions to educate parents of male adolescents about the importance of HPV vaccination for males.

We found that the proportion of parents with children of all racial and ethnic groups who were HPV vaccine hesitant declined significantly from 2010 to 2012 but then remained stable from 2012 to 2022. Although this reflects a concerning trend across all groups, this persistence in vaccine hesitancy may be especially concerning for racial and ethnic minority groups, given their higher burden of some HPV‐associated cancers, such as a higher incidence of cervical cancer and HPV‐associated oropharyngeal cancers among Hispanic and non‐Hispanic Black populations as well as higher mortality rates compared to non‐Hispanic Whites.^28^, ^29^, ^30^ The stable trend among parents of Hispanic adolescents could possibly be due to cultural, language and linguistic barriers, lack of awareness about HPV and HPV vaccine and HPV‐associated cancers, lack of racial concordance between Hispanic parents and providers, or possible mistrust in the health care system. For parents of non‐Hispanic Whites, and non‐Hispanic Blacks, the stagnant vaccine hesitancy may also be influenced by lack of awareness about HPV and HPV vaccine, concerns regarding sexuality, safety and low perceived risk of their daughters contracting HPV, and the perception that the vaccine is unncecsary.^23^, ^31^, ^32^ It should be noted that adolescents with Hispanic parents have the lowest rates of HPV vaccine hesitancy across the different race/ethnicity groups whereas adolescents with non‐Hispanic White parents have the highest stable rates. Future studies should explore why non‐Hispanic White parents have higher vaccine hesitancy rate than other racial/ethnic groups.

There were two additional noteworthy findings regarding HPV vaccine hesitancy. First, although patterns of change in hesitancy over time were similar across married and unmarried mothers, married mothers were more likely to be vaccine hesitant than unmarried mothers. Prior studies have shown mixed resulted on vaccination and marital status, with some finding greater vaccine acceptance among unmarried adults whereas others found the reverse or no effect of marital status.^33^, ^34^, ^35^ Our results further indicate a potentially complex relationship between marital status and vaccination and highlight a need for investigation into the influence of marital partners on HPV vaccine acceptance among parents. Second, mothers of adolescents living above the poverty line were more likely to be vaccine hesitant than those living below the poverty line. This finding may seem unexpected, given vaccination disparities are often observed among families living in poverty. However, the link between socioeconomic status and vaccination is complex and multifaceted. For example, financial barriers and lack of accessibility are also significant contributors to vaccine uptake.^36^, ^37^ Furthermore, some research suggests a potential “privilege paradox,” where higher socioeconomic is associated with greater vaccine hesitancy, perhaps due to factors such as strong healthism beliefs.^38^, ^39^, ^40^, ^41^ Our study provides additional evidence of a paradoxical effect of socioeconomic status that should be explored in future research.

There was an initial decrease in HPV vaccine hesitancy from 2010 to 2012 for all characteristics examined in the study, but rates of hesitancy remained stable from 2013 to 2022 for most of them. One possible explanation for stable levels of vaccine hesitancy could be that targeted interventions, programs, and campaigns designed to reduce vaccine hesitancy and increase HPV vaccine uptake no longer seem to be effective despite having an initial positive effect. Previous studies have identified several possible reasons for vaccine hesitancy. Most hesitancy revolves around misconception, fear, or mistrust.^42^ Other reasons include a desire for autonomy and other moral concerns.^42^ Theories of behavior change assume/suggest that attitudes like vaccine trust are drivers of vaccination motivation and behavior, and influencing these attitudes could change motivation and intentions.^43^ Trust in vaccination is a conceptually broad concept that constitutes attitudes or beliefs regarding the efficacy and safety of the vaccine and that it is being provided by a trustworthy medical system. Public health and medical authorities prioritize improving vaccine confidence, particularly as vaccine hesitant parents are more likely to be exposed to vaccine misinformation, damaging their trust in vaccines. Providers are trusted sources of vaccine information and can play a paramount role in improving parents’ confidence in HPV vaccination for their children.^44^ Thus, health care providers should recommend the HPV vaccine to all eligible individuals and address fears and concerns of teens and their parents. Further research is needed to understand the factors contributing to these stable patterns and to design tailored interventions that maintain or reinvigorate progress among these subgroups.

### Strengths and limitations

Strengths of this study include the use of a nationally representative data set, and thus the findings are generalizable to parents and adolescents living in the United States. Despite the strengths, the study findings should be interpreted in the context of these limitations. First, the cross‐sectional nature of the data did not allow us to assess for causation; thus, our results only describe associations based on characteristics over the years. Second, parents were not followed over time to see if their hesitancy has changed but rather a different group of patients was used each year. Furthermore, there is the possibility of nonresponse bias, although the use of weighting by NIS–Teens potentially reduces this bias. It is possible that vaccine‐hesitant parents who are leery of research may have refused to respond to the NIS–Teen survey, which could suggest that our estimate of vaccine hesitancy may be lower than the rate in the population. Last, there is the possibility of recall bias as the hesitancy question was asked only of parents who reported no vaccination receipt, thus findings do not reflect parents who vaccinated their child, but the timing may have been delayed due to initial hesitancy.

### Public health implications

HPV vaccination rates still remain below the Healthy People 2030 goal of attaining 80% completion for adolescents.^11^ In the United States, lack of parents’ intention to vaccinate their teens remains a major public health problem and addressing vaccine hesitancy may aid in increasing the suboptimal coverage of HPV vaccination.^19^ Because vaccine hesitant parents are more vulnerable to vaccine misinformation that could destroy their trust toward the vaccine,^45^ public health officials and health care systems need to prioritize addressing vaccine misconceptions and disinformation. Indeed, research has shown that parents who are exposed to HPV vaccine misinformation on social media are less likely to vaccinate their children.^46^ Enforcing trust in medical providers is essential in boosting parents’ confidence in the HPV vaccine.^44^ Findings from this study may aid planning and implementing targeted and tailored interventions and policy changes that could encourage and promote HPV vaccine uptake and decrease hesitancy to help reach the Healthy People 2030 goal of 80% completion rate.

In conclusion, our study highlights the demographic characteristics of over a decade long trend in vaccine hesitancy. We found that rates of vaccine hesitancy decreased between 2010 and 2012 but remained stagnant between 2012 and 2022. Understanding the trends is imperative for the development of targeted interventions to improve vaccination rates in those whose parents are vaccine hesitant. This study emphasizes the need to dispel misconceptions and create awareness about HPV vaccine across all strata of the society. The data used in this study covered part of COVID‐19 pandemic, but future studies should examine the effects of the pandemic on vaccine hesitancy.

## AUTHOR CONTRIBUTIONS

**Dina K. Abouelella:** Conceptualization; data curation; formal analysis; investigation; methodology; validation; visualization; writing—original draft. **Mrudula Nair:** Conceptualization; data curation; investigation; methodology; validation; visualization; writing—original draft. **Mary A. Gerend:** Data curation; formal analysis; investigation; methodology; validation; writing—original draft; writing—review and editing. **Olivia Aspiras:** Data curation; investigation; methodology; validation; writing—review and editing. **Nosayaba Osazuwa‐Peters:** Conceptualization; data curation; investigation; methodology; validation; writing—review and editing. **Eric Adjei Boakye:** Conceptualization; data curation; formal analysis; investigation; methodology; supervision; validation; visualization; writing—original draft, writing—review and editing.

## CONFLICT OF INTEREST STATEMENT

Nosayaba Osazuwa‐Peters reports consulting fees from Merck and Navigating Cancer; and grant and/or contract funding from National Institutes of Health. The other authors declare no conflicts of interest.

## Supporting information

Table S1

## Data Availability

The data that support the findings of this study are openly available in NIS–Teens at https://www.cdc.gov/nchs/nis/data_files_teen.htm.
