# Supplementary material for: Trends in sociodemographic factors associated with human papillomavirus vaccine hesitancy in the United States: 2010–2022
Source: Cancer. 2026 Sep 28;132(19):e70636. doi: 10.1002/cncr.70636 (PMC13619007; doi:10.1002/cncr.70636)
Supplement: Supplementary file 1 [file CNCR-132-e70636-s001.pdf]

Table 1. Characteristics of study respondents: National Immunization Survey – Teen, 2010-2022, (unweighted n=218,335)

	**Overall**
Adolescent	
Age, in years	
13	45093 (22.1)
14	42911 (21.0)
15	40211 (19.7)
16	39559 (19.4)
17	36422 (17.8)
Sex	
Female	82898 (40.6)
Male	121298 (59.4)
Race/ethnicity	
Non-Hispanic White	135395 (66.3)
Non-Hispanic Black	19441 (9.5)
Hispanic	29546 (14.5)
Non-Hispanic Other	19814 (9.7)
Poverty status	
Above poverty	166355 (86.8)
Below poverty	25212 (13.2)
Number of doctor's visits in the past 12 months	
>= Four	35684 (17.6)
Two – three	68655 (33.9)
One	61829 (30.5)
None	36621 (18.1)
Census region	
Northeast	34401 (16.8)
Midwest	44201 (21.6)
South	80043 (39.2)
West	45551 (22.3)
Provider Recommendation	
Yes	70835 (36.0)
No	125662 (64.0)
Mother	
Age, in years	
≤ 34 years	15192 (7.4)
35 to 44 years	88295 (43.2)
≥ 45 years	100709 (49.3)
Marital status	
Married	150823 (73.9)
Not married	53373 (26.1)

Education	
College graduate	92593 (45.3)
Some college	58868 (28.8)
High school Graduate	35886 (17.6)
Less than high school	16849 (8.3)
Number of children under age 18	
One	81386 (39.9)
Two or three	102184 (50.0)
Four or more	20626 (10.1)
